# Monitoring mosquitoes in urban Dar es Salaam: Evaluation of resting boxes, window exit traps, CDC light traps, Ifakara tent traps and human landing catches

**DOI:** 10.1186/1756-3305-4-40

**Published:** 2011-03-21

**Authors:** Nicodem J Govella, Prosper P Chaki, John M Mpangile, Gerry F Killeen

**Affiliations:** 1 Ifakara Health Institute, Coordination Office, PO Box 78373, Kiko Avenue, Mikocheni, Dar es Salaam, United Republic of Tanzania; 2 Liverpool School of Tropical Medicine, Pembroke place, Liverpool L3 5QA, UK; 3 Salvation Army of Tanzania, Monitoring and Evaluation Department, P.O. Box 1273, Kilimanjaro, United Republic of Tanzania

## Abstract

**Background:**

Ifakara tent traps (ITT) are currently the only sufficiently sensitive, safe, affordable and practical method for routine monitoring host-seeking mosquito densities in Dar es Salaam. However, it is not clear whether ITT catches represent indoors or outdoors biting densities. ITT do not yield samples of resting, fed mosquitoes for blood meal analysis.

**Methods:**

Outdoors mosquito sampling methods, namely human landing catch (HLC), ITT (Design B) and resting boxes (RB) were conducted in parallel with indoors sampling using HLC, Centers for Disease Control and Prevention miniature light traps (LT) and RB as well as window exit traps (WET) in urban Dar es Salaam, rotating them thirteen times through a 3 × 3 Latin Square experimental design replicated in four blocks of three houses. This study was conducted between 6^th ^May and 2^rd ^July 2008, during the main rainy season when mosquito biting densities reach their annual peak.

**Results:**

The mean sensitivities of indoor RB, outdoor RB, WET, LT, ITT (Design B) and HLC placed outdoor relative to HLC placed indoor were 0.01, 0.005, 0.036, 0.052, 0.374, and 1.294 for *Anopheles gambiae sensu lato *(96% *An. gambiae s.s *and 4% *An. arabiensis*), respectively, and 0.017, 0.053, 0.125, 0.423, 0.372 and 1.140 for *Culex spp*, respectively. The ITT (Design B) catches correlated slightly better to indoor HLC (r^2 ^= 0.619, P < 0.001, r^2 ^= 0.231, P = 0.001) than outdoor HLC (r^2 ^= 0.423, P < 0.001, r^2 ^= 0.228, P = 0.001) for *An. gambiae s.l*. and *Culex spp *respectively but the taxonomic composition of mosquitoes caught by ITT does not match those of the indoor HLC (χ^2 ^= 607.408, degrees of freedom = 18, P < 0.001). The proportion of *An. gambiae *caught indoors was unaffected by the use of an LLIN in that house.

**Conclusion:**

The RB, WET and LT are poor methods for surveillance of malaria vector densities in urban Dar es Salaam compared to ITT and HLC but there is still uncertainty over whether the ITT best reflects indoor or outdoor biting densities. The particular LLIN evaluated here failed to significantly reduce house entry by *An. gambiae s.l*. suggesting a negligible repellence effect.

## Background

In urban Dar es Salaam, Tanzania the principal malaria vectors are species of *An. gambiae *complex and *An. funestus *[1]. *Culex spp*, is also present in larger numbers [1], causing appreciable nuisance and is known to transmit Lymphatic Filariasis [2-6]. Human infection with *Wuchereria bancrofti *was generally thought to be increasing in urban African communities due to rapid urbanization coupled with inadequate sanitary facilities which provide ideal breeding habitats [7] for mosquitoes in the *Culex pipiens *complex (*Culex pipiens *L *Culex quinquefasciatus*,) [8], which is a major vector of lymphatic filariasis in South Asia, East Africa and Americas particularly in urban areas [3,9-14]. Although recent global effort to eliminate the filarial infections through mass drug administration (MDA) has reversed this trend to some degree [15-17], it is becoming increasingly clear that elimination of filariasis transmission is unlikely to be achieved by MDA alone [5,18] so vector control remains an option to be considered that will necessitate routine monitoring of vector densities.

In its initial stages, routine monitoring of adult mosquito densities by the Dar es Salaam Urban Malaria Control program (UMCP) was only possible with the laborious, uncomfortable, requiring intensive supervision and potentially hazardous human landing catch (HLC) for several years [1,19]. The Dar es Salaam Urban Malaria Control program relies on weekly application of larvicides to all potential breeding habitats observed by community-based staff assigned to defined areas of approximately 0.6 km^2 ^[19-21]. In order to enable effective management of routine larvicide application activities, the adult mosquito surveillance system for this programme needs to report mosquito densities at correspondingly high spatial and temporal resolution. This prompted development and evaluation of a safe, sensitive, cheap, practical and affordable alternative to HLC that allows intensive and extensive monitoring of malaria vectors. A series of Ifakara Tent Trap (ITT) designs have been tried and the B design has proven efficacious [22] and effective [23] in terms of both number and species composition of mosquitoes caught. It is also cost-effective relative to other sampling methods in terms of cost per mosquito trapped [23]. The B design exposed human subjects to mosquito bites while emptying the large trap chamber [22,23], this model has since been modified to circumvent this problem, but the new design (C) was not available at the time of this study [24]. The ITT appears to be the most promising method for routine surveillance of biting densities of host-seeking mosquitoes in this setting and may be useful in a variety of African settings.

While monitoring host-seeking mosquito densities are an essential part of understanding disease, samples of resting mosquitoes [25] are also required to enable assessment of host feeding patterns through blood meal analysis [26-28]. The proportion of blood meals that each vector species obtains from humans is a critical determinant of, not only transmission intensity, but also the efficacy of interventions targeted at humans or the houses they live in [29-35]. Sample of resting mosquitoes for blood meal analyses are therefore important for selecting appropriate control strategies, particularly as vector population composition may become dominated by zoophagic species once high coverage by insecticide-treated nets [36-38] or indoor residual spraying [39-41] is achieved. ITT and HLC both primarily sample host-seeking mosquitoes [22-24] so either resting collection techniques [42-44] or window exit traps (WET) [44] are required to effectively characterize the feeding behaviours of vector mosquitoes that are relevant to intervention efficacy and selection.

The WET has been found useful for monitoring malaria vector density trends in Southern Africa [45,46], Equatorial Guinea [47] and for vectors of Japanese encephalitis [48] in Korea. However, their sensitivity is likely to be site-specific and strongly influenced by house design. Resting boxes were found to be highly selective in sampling specific mosquito species in coastal areas of the United States of America [49], but have also shown potential for monitoring *Culex quinquefasciatus *and *Aedes aegypti *in urban Brazil [50].

This article therefore presents an assessment of a number of mosquito trapping methods compared with HLC catches in Dar es Salaam, including the widely used Centers for Disease Control and Prevention miniature Light Trap (LT) and the WET design commonly used in programmatic contexts, in a rigorous formal comparison for the first time in this urban setting. We also assessed whether catches with the B design of the ITT best represent the indoor or outdoor fractions of mosquito populations because, although this device is placed outdoors, it does resemble a small house and requires the mosquito to enter it so it may selectively sample indoor-biting mosquitoes.

## Methods

### Study site

This study was conducted at Mchikichini and Jangwani wards situated along the edge of Msimbazi River Valley in urban Dar es Salaam, the largest and most economically important city in Tanzania. The city is located at the shores of Indian Ocean coast with humid and hot climatic condition [43]. There are classically two rainy seasons: a main rainy season from March to June and a shorter, less intense rainy season from October to December [1].

Dar es Salaam is also the home of the UMCP, a community-based vector control programme which primarily implements locally-supervised larviciding applied on a weekly basis at the neighbourhood level with vertical oversight from the city council [1,19-21,23,51-56]. *An. gambiae *sibling species can grow from egg to adult in one week or less [33,57] so the adult mosquito surveillance system for this programme needs to be, not only affordable and practical [23], but also both spatially and temporally intensive to detect coverage gaps as they occur on such fine geographic scales as neighbourhoods, housing clusters and even individual plots [20,21] on a weekly or even daily basis [19,58]. The need for sensitive adult malaria mosquito surveillance in this setting is compounded by low levels of local malaria transmission and correspondingly sparse vector populations that mediate it.

Transmission of malaria in urban Dar es Salaam is generally low with an entomologic inoculation rate of about one or less infectious bite per person per year [19], corresponding to the approximate limit of detection of malaria transmission by most entomological surveillance systems [59]. Members of the *Anopheles gambiae *complex (*An. gambiae sensu stricto*, *An. arabiensis*, *An. merus*) and *An. funestus *are the primary malaria vector in this setting, with *An. gambiae s.s*. and *An. arabiensis *being most important [1]. While the nightly biting peak of *An. gambiae s.s*. in Dar es Salaam is consistent with that of classical reports [33], recent observations show that this vector species, together with *An. arabiensis*, tends to bite predominantly outdoors [1,60].

### Trapping methods

#### Resting boxes (RB)

Resting boxes made of cardboard with one open end and black cotton cloth lined inside them [23], were each placed indoor in a room occupied by a person and outdoor in a shaded area. Mosquitoes caught were retrieved from the boxes using a hand-held aspirator from 8.00 am to 9.00 am on the morning following each sampling night.

#### Window exit trap (WET)

**Figure 1:**
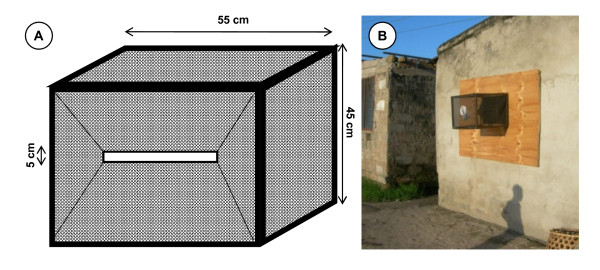
**Window exit trap before fixing to a house window (**A**), and after installation (**B**)**.

Window exit traps are rectangular boxes made of a wooden frame covered in Teflon^®^-coated woven fiberglass netting, with a slit-shaped rectangular tilted wire opening at one side as a mosquito entrance (Figure 1A) and a sealable cotton sleeve aspirator inlet on the other side. The trap is first attached to a plywood sheet with screws and then the board plus trap combination is screwed to a house window frame (Figure 1B). Note that the edges of the plywood were wrapped with a foam seal to cover the gap between the board and the wall of the house, as well as protecting the wall from being scratched by the board. The traps were installed only to houses without intact screens or houses whose owners provided written informed consent to remove the screen under condition of being compensated with free installation of new screening at the end of the study. Mosquitoes were retrieved from the trap using hand-held aspirator through a sealable sleeve from 8.00 am to 9.00 am.

#### Centers for Disease Control and Prevention miniature light traps (LT)

CDC miniature light traps (model 512) with inflorescent bulbs were each hung inside a house near an occupied bed covered with either an untreated net or a long lasting insecticidal net (LLIN), with one block in each location assigned to the two types of nets to test for the effect of net treatment upon LT trap efficiency. The Permanet 2.0^® ^brand of LLIN was used. The trap was hung approximately 150 cm from the floor surface and placed with the pan touching one side of the net at the end where the occupant's feet lay [61].

#### Ifakara tent trap (ITT Design B)

The B design of the ITT was placed approximately 5 m outside the house with a team member sleeping inside it and mosquitoes were collected in the morning as previously described [22,23]. The C design of ITT [24] was not used because it had not been developed and evaluated at the time.

#### Human landing catch (HLC)

To conduct HLC, each adult male collector exposed his lower limbs and collected the mosquitoes when they landed on his legs with an aspirator [1,27,44,62,63]. HLC was conducted by a single catcher at each station for 45 minutes each hour, allowing 15 minutes break for rest. To obtain full hourly biting densities, the catches for each hour were therefore divided by 0.75 [1]. Collections were conducted both indoors and outdoors in accordance with the experimental design described below.

### Experimental design

**Figure 2:**
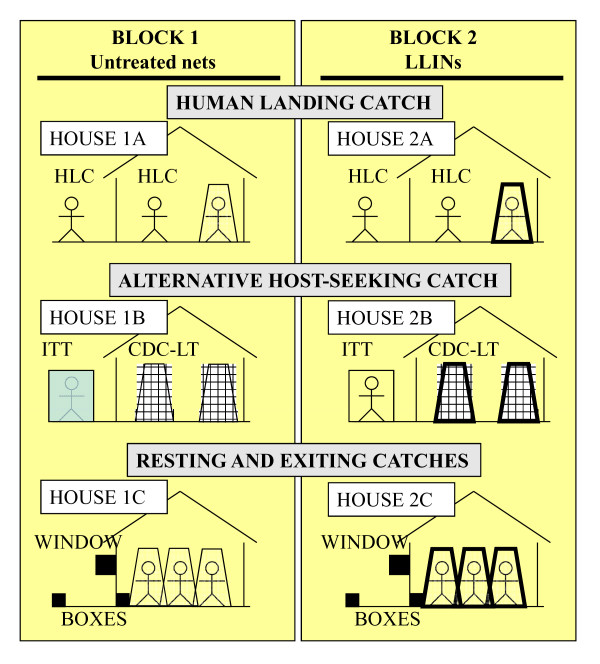
**Schematic illustration of a typical night's experimental set up (arrangement 1 as illustrated in figure 3) at one location with two blocks, one of which has occupants using untreated nets while the other has participants using long-lasting insecticidal nets (LLINs)**.

**Figure 3:**
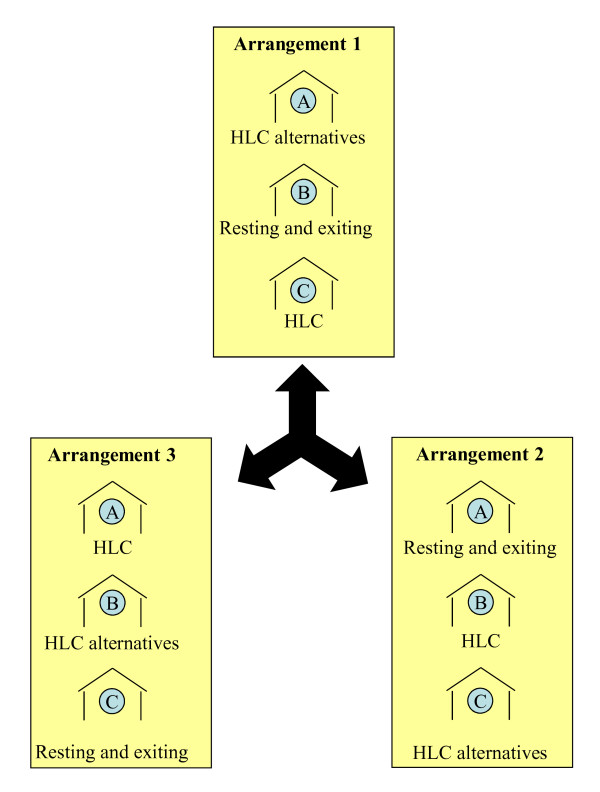
**Schematic presentation of three possible arrangements of trapping methods rotated in order through the three houses in any given block**. Note that the letters in blue circle represent the identifier within the block for each house, specified as 1A, 1B and 1C for houses in block 1 and 2A, 2B and 2C in for houses in block 2.

Four blocks (two from Mchikichini ward and the remaining two from Jangwani ward) of three houses each, with correspondingly matched outdoor catching stations about 5 m away from each house were selected. Only houses with open eaves and without ceiling boards, made of blocks with plastered walls and corrugated iron roofs, were chosen and they were distributed approximately 50 m apart. In each location, the two blocks were set up so that one block had all participants protected with untreated nets while those in the other slept under LLINs. As described in the section entitled *Ethical clearance and protection of human participants*, these were planned based on the existing ownership of nets so that participants only experienced either no change or an increase in protection against mosquitoes and malaria: only participants lacking a net were provided with an untreated net and participants already owning a net of any description were provided with an Perma Net 2.0^® ^LLIN (Polyester treated with deltamethrin, developed by Vastergaard Frandsen A/S Kolding, Denmark) [64-66]. The resting box, ITT design B, and HLC were placed in the corresponding respective indoor stations so that the combined indoor and outdoor stations at each house within each block could be considered to represent the conventional HLC gold standard, alternative host-seeking catching methods or methods for sampling resting and house-exiting mosquitoes, respectively (Figure 2). These indoor-outdoor combinations were rotated through all three houses of each block (Figure 3) for a total of thirteen complete rotations in 3 × 3 Latin Square experiment design. This experiment was conducted between 6^th ^May and 2^rd ^July 2008 period, corresponding to the main rainy season and annual peak mosquito biting densities [52]. Mosquitoes were collected from 19.00 to 08.00 h each night.

### Processing of Samples

All *Anopheles *mosquitoes caught were sorted and morphologically identified [57] with the aid of a stereomicroscope in the field. A total of 1180 *An. gambiae s.l *from all traps, were stored in tubes with desiccated silica for subsequent identification to sibling species level by polymerase chain reaction [67]. All *Culex *were counted, categorized as male or female and discarded.

### Data analysis

#### Sensitivity differences among trapping methods

Data analyses were computed using SPSS version 16.0 for Microsoft Windows (SPSS Inc., Chicago IL). Generalized estimating equations (GEE) were employed to assess the influence of trap type upon mosquito catches by treating house as subject variable with trap type-indoor/outdoor assignment combination and date as within-subject variables. Catches for female *An. gambiae s.l*. and *Culex spp*, were each treated as the dependent variable in separately fitted models. Normal distribution with a natural logarithm link function and exchangeable working correlation matrix were selected for these dependent variables. In the first place for fitting to the catches of *An. gambiae s.l*., all trap types were included in the model, but yielded inestimable parameter values for both indoor and outdoor resting boxes so these two methods were thereafter removed from the fitted dataset.

#### The distribution of mosquito taxa among sampling methods and correlation of the catches

Trap type may affect taxonomic composition of mosquito catches [68] so the influence of trapping method upon the distribution of mosquitoes was analyzed by χ^2 ^test [69]. Comparison of multiple pair-wise Pearson correlation tests using logarithmically transformed data (log_10 _(*x*+1) for *An. gambiae s.l*. and log_10 _(*x*) for *Culex spp *of female catches aggregated by date was used to test whether the catches by the ITT best represent the indoor or outdoor biting catches.

#### The effect of net type on the indoor versus outdoor distribution of mosquitoes

The only method which yielded sufficient numbers of *An. gambiae s.l*. and for which both indoor and matching outdoor catches in the same house and night were available was HLC. Comparing the effect of LLINs versus untreated nets upon catches was therefore only possible for this particular method. All mosquitoes caught with HLC in a given house and on a particular night were either caught indoors or outdoors, hence the distributions of *An. gambiae s.l*. with regards to net type was analyzed by binary logistic regression, treating indoor versus outdoor catches of *An. gambiae s.l*. as binary outcome.

### Ethical clearance and protection of human participant

Ethical approval was obtained from Institutional review board of Ifakara Health Institute in Tanzania (IHI/IRB/No. A50) and Medical Research Coordination Committee of the National Institute of Medical Research in Tanzania (NIMR/HQ/R. 8c/Vol. ii/03) and the Ethics Committee of the Liverpool School of Tropical Medicine in the UK (09.60). Written informed consent describing the potential risks and benefits of the study was obtained from all study participants before commencing the study and re-confirmed on each experimental night. Volunteers were screened for malaria parasites by microscopy during recruitment and on a weekly basis throughout the experiment. Those who were found malaria positive were offered treatment free of charge with Artemether-Lumefantrane (Co-Artem^®^, Roche, Basel, Switzerland), the recommended first-line treatment for malaria in the United Republic of Tanzania. The untreated net versus LLIN blocks were assigned so that no individual participant who already had a net was provided with an untreated net to replace it: participants lacking a net were provided either an untreated net or an LLIN while individuals with an existing net, untreated or otherwise, were all provided with a free Perma Net^® ^2.0 LLIN.

## Results

### Sensitivity of alternative traps relative to indoor human landing catch

The number of mosquitoes caught by each collection method is shown in Table 1. The RB, WET, and the LT caught far less *An. gambiae s.l*. than the indoor HLC reference method. ITT was the only alternative method that caught useful numbers of this vector complex (Table 1), with approximately one quarter the sensitivity of indoor HLC (Table 2). The LT, however, appeared relatively sensitive for sampling *Culex spp*, exceeding even the number caught by the ITT (Table 1 and 2). All alternative trapping methods, with the exception of the outdoor HLC, sampled significantly less *An. gambiae s.l*. than the indoor HLC reference method (Table 2). The outdoor HLC, caught as many *An. gambiae s.l*. and significantly more *Culex spp *than the indoor HLC reference method (Table 2).

**Table 1:** Number of mosquitoes caught by different methods and crude estimates of sensitivity relative to indoor human landing catch

**Collection methods**	**Trap night**	**Total catch**	**Mean catch**	**Relative sensitivity**
				
*Anopheles gambiae s.l*.				
				
Resting boxes indoor	156	6	0.038	0.01
Resting boxes out	156	3	0.019	0.005
Window trap	156	21	0.135	0.036
CDC light trap	155	30	0.194	0.052
Ifakara tent trap	156	216	1.385	0.374
HLC outdoor	156	748	4.795	1.294
HLC indoor	156	578	3.705	NA
				
*Anopheles funestus*				
				
Resting boxes indoor	156	0	0	0
Resting boxes outdoor	156	0	0	0
Window trap	156	0	0	0
CDC light trap	155	0	0	0
Ifakara tent trap	156	1	0.006	0.158
HLC outdoor	156	19	0.122	3.210
HLC indoor	156	6	0.038	NA
				
*Anopheles zeimanii*				
				
Resting boxes indoor	156	4	0.03	0.017
Resting boxes outdoor	156	2	0.01	0.005
Window traps	156	2	0.01	0.005
CDC light traps	155	2	0.01	0.005
Ifakara tent traps	156	9	0.06	0.033
HLC outdoors	156	460	2.95	1.629
HLC indoors	156	283	1.81	NA
				
*Culex spp*				
				
Resting boxes indoor	156	293	1.878	0.017
Resting boxes outdoor	156	931	5.968	0.053
Window traps	156	2208	14.153	0.125
CDC light traps	155	7435	47.968	0.423
Ifakara tent traps	156	6585	42.212	0.372
HLC outdoors	156	20163	129.250	1.140
HLC indoors	156	17688	113.385	NA

NA = not applicable because this is a reference method

**Table 2:** Mosquito sampling sensitivity of alternative traps relative to the indoor human landing catch as determined using generalized estimating equations

**Collection methods**	**RR [95%CI]**	***P value***
		
*Anopheles gambiae s.l*.		
		
Resting boxes indoor	NE	NE
Resting boxes outdoor	NE	NE
Window exit trap	0.01 [0.002, 0.034]	< 0.001
CDC light trap	0.02 [0.009, 0.032]	< 0.001
Ifakara tent trap	0.26 [0.208, 0.330]	< 0.001
HLC outdoor	1.07 [0.851, 1.356]	0.549
HLC indoor	1.00*	NA
*Culex spp*		
		
Resting boxes indoor	0.02 [0.010, 0.026]	< 0.001
Resting boxes outdoor	0.07 [0.020, 0.274]	< 0.001
Window exit trap	0.11 [0.077, 0.166]	< 0.001
CDC light trap	0.50 [0.280, 0.893]	0.019
Ifakara tent trap	0.34 [0.256, 0.461]	< 0.001
HLC outdoor	1.17 [1.077, 1.278]	< 0.001
HLC indoor	1.00*	NA

RR = relative rate, CI = confidence interval, NE = not estimable

NA = not applicable because this is a reference method

* Reference value

### Sibling species composition of An. gambiae sensu lato

Respectively, 96% (871) and 4% (41) of 912 (7, 10, 22, 94 and 779 Sub sample from RB, WET, LT and HLC respectively) successfully amplified specimens *of An. gambiae s.l*. were *An. gambiae s.s*. and *An. arabiensis*. This implies that the results presented here overwhelmingly reflect the response of *An. gambiae s.s*. to these traps.

### Effect of sampling technique upon taxonomic composition of female mosquito and correlation of catches

**Figure 4:**
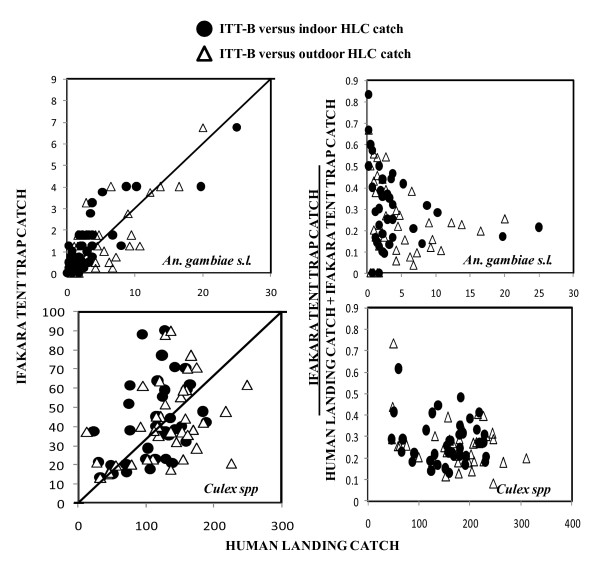
**Correlation and density dependence of Ifakara tent trap (ITT) **[22,23]**model B sampling efficiency relative to human landing catch (HLC)**. The correlation between the catches of *An.gambiae s.l*. and *Culex spp *with ITT and HLC plotted in absolute number is presented in left panels with complete equivalence depicted by the diagonal line. Right panels illustrate density dependence as catches in ITT divided by the sum catches of ITT and HLC against the absolute catches of the HLC reference method.

*An. gambiae s.l*. (2.78%), *An. funestus *(0.05%), *An. ziemanii *(1.32%) and *Culex spp *(95.85%) were the only mosquitoes captured in this study. Trap type significantly affected the composition of catches (χ^2 ^= 607.408, degrees of freedom = 18, P < 0.001). Apart from such an overall χ^2 ^all pair-wise χ^2 ^comparisons of either outdoor HLC or any of the alternative methods with indoor HLC proved significant (P ≤ 0.0001). As illustrated in Figure 4 the catches of *An. gambiae s.l*. and *Culex spp *by ITT correlated consistently slightly better with those of the indoor HLC (r^2 ^= 0.619, P < 0.001 and r^2 ^= 0.304, P = 0.001, respectively) than the outdoor HLC (r^2 ^= 0.423, P < 0.001 and r^2 ^= 0.228, P = 0.001, respectively). However, this pattern does not hold consistently when previous field studies of the same trap design are considered: Correlation results from the previous study in a rural setting for *An. gambiae s.l*. were (r^2 ^= 0.162 P = 0.098 and r^2 ^= 0.462, P = 0.002 for ITT versus indoor and outdoor HLC, respectively) and (r^2 ^= 0.452, P = 0.002 and r^2 ^= 0.260, P = 0.033 for *Culex spp *by ITT versus indoor and outdoor HLC, respectively.

### Effect of long-lasting insecticidal nets upon mosquito sampling

Human landing catch was the only method for which sufficient numbers of *An. gambiae s.l*. were caught to assess the impact of LLINs upon the relative fraction of mosquitoes found inside or outside houses. There was no significant difference in the proportion of *An. gambiae s.l*. caught indoors between LLINs and untreated bed nets houses (Table 3).

**Table 3:** The effect of treatment on proportion of *An. gambiae s.l*. sampled indoor and outdoor determined by logistic regression as described in the method sections.

**Categorical variables**	***An. gambiae s.l*. caught indoor (%)**	**OR[95%CI]**	***P***
			
Treatment			
			
Long lasting net	44.94 (333/741)	1.13 [0.91, 1. 41]	0.265
Untreated bed net	41.88 (245/340)	1.00*	NA

NA = not applicable because this is a reference group

OR = odds ratio, CI = Confidence interval, 1.00*= reference value

## Discussion

Sustained control of pathogen- transmitting mosquitoes requires sensitive and representative surveillance. This study compares a wide range of trapping methods, and demonstrates very poor performance of the RB, WET and LT for sampling adult malaria mosquitoes. This implies that such tools are not appropriate for surveillance and monitoring the impact of mosquito control measures in this urban setting where the UMCP targets relatively sparse populations of *Anopheles *malaria vectors. These results also confirm the previous observational reports that the LT has very low sensitivity in this urban setting [19]. This cannot be explained by the observation that *An. gambiae *is exophagic in this setting [1,60] because the reference HLC method was also conducted indoors. While no particular explanation is obvious for such surprisingly poor performance by LT, we speculate that the light source from the LT, which is thought to play a vital role in attracting mosquitoes [70], may have competed poorly for the attention of *Anopheles *in this highly illuminated [71], urbanized environment.

Some reports have suggested that the RB baited with urine odour are useful [72] for surveillance of *An. arabiensis*, the most exophilic [73] sibling species of the *An. gambiae s.l*. complex [57,74] in lower Moshi, Tanzania. However, this conclusion was neither supported by this study nor by a previous effectiveness evaluation in Dar es Salaam [23] which relied on unbaited RB. While these results are discouraging, it may be possible to improve the sensitivity of the approach by lining the boxes internally either with a sticky surface [75] or a collapsible collection bag to maximize the catch size, because we observed that mosquitoes which entered the RB often escaped, particularly during retrieval.

Likewise the weak performance by WET can be possibly partly explained by the architectural of the local houses. Most houses used in this study apart from having open eaves and lacking a ceiling, also had walls separating adjacent rooms which did not reach the roof. It was therefore likely that many mosquitoes which entered a room fixed with WET exited via other rooms without a WET. Nevertheless, without such ready exits, there is also limited opportunity for mosquitoes to enter houses in the first place so there may be a fundamental a limit to how efficacious such exit traps can be outside of experimental huts. Furthermore, variations in housing design are a normal feature of representative mosquito sampling so these disappointing results should be interpreted at face value until proven otherwise. It should also be noted that while this approach has been applied and advocated in a number of programmatic settings [45-47,76], to our knowledge this is the first time the efficacy of this trapping method has been formally evaluated in comparison with HLC or other standard methods in typical residences rather than in experimental huts.

The correlation results obtained from this study indicated the catches from ITT relate better to those from the indoor rather than outdoor HLC but the taxonomic composition of female mosquitoes caught by ITT does not match those of the indoor HLC and re-analysis of data obtained from the previous study in rural setting [22], yield contradictory correlation results that although consistent with this study for *Culex spp *the reverse was observed for the *An. gambiae s.l*. population, consisting primarily of *An. arabiensis*, in that study. It therefore remains unclear whether densities measured by ITT best reflect indoor or outdoor catches.

Consistent with our previous study of ITT evaluation [22,23] it appears that this trap has potential for both research and routine programmatic surveillance applications.

Although, pyrethroid treated bed nets are commonly thought to reduce house entry by *An. gambiae s.l*. [77-80], the particular LLIN product evaluated in this study failed to significantly deter *An. gambiae s.l*. from entering local houses. This observation is consistent with experimental hut studies in other parts of Tanzania [64,65]and Benin [81]. We conclude that, in this urban Tanzanian setting, negligible protection against malaria transmission exposure can be expected for non-users sharing the same house. This observation implies also that the reliability of human landing catches in estimating indoor *An. gambiae s.l*. catches in this setting is not affected by the presence of this particular brand of LLINs.

## Competing interests

The authors declare that they have no competing interests.

## Authors' contributions

NJG designed and implemented mosquito sampling protocol in collaboration with other authors. He supervised the data collection, performed analysis, interpreted the results and drafted the manuscript in consultation with other authors. PPC, JP supported the design and implementation of the study. GFK supervised the design, implementation of the study, data analysis and drafting of the manuscript. All authors read and approved the final manuscript.
